# Older adults recover more marginal knowledge and use feedback more effectively than younger adults: evidence using “I don’t know” vs. “I don’t remember” for general knowledge questions

**DOI:** 10.3389/fpsyg.2023.1145278

**Published:** 2023-05-31

**Authors:** Sharda Umanath, Talia E. Barrett, Stacy Kim, Cole A. Walsh, Jennifer H. Coane

**Affiliations:** ^1^Department of Psychological Science, Claremont McKenna College, Claremont, CA, United States; ^2^Department of Psychology, Colby College, Waterville, ME, United States

**Keywords:** aging, accessibility, availability, marginal knowledge, retrieval failures, feedback

## Abstract

Through three experiments, we examined older and younger adults’ metacognitive ability to distinguish between what is not stored in the knowledge base versus merely inaccessible. Difficult materials were selected to test this ability when retrieval failures were very frequent. Of particular interest was the influence of feedback (and lack thereof) in potential new learning and recovery of marginal knowledge across age groups. Participants answered short-answer general knowledge questions, responding “I do not know” (DK) or “I do not remember” (DR) when retrieval failed. After DKs, performance on a subsequent multiple-choice (Exp. 1) and short-answer test following correct-answer feedback (Exp. 2) was lower than after DRs, supporting self-reported *not remembering* reflects failures of accessibility whereas *not knowing* captures a lack of availability. Yet, older adults showed a tendency to answer more DK questions correctly on the final tests than younger adults. Experiment 3 was a replication and extension of Experiment 2 including two groups of online participants in which one group was not provided correct answer feedback during the initial short-answer test. This allowed us to examine the degree to which any new learning and recovery of access to marginal knowledge was occurring across the age groups. Together, the findings indicate that (1) metacognitive awareness regarding underlying causes of retrieval failures is maintained across different distributions of knowledge accessibility, (2) older adults use correct answer feedback more effectively than younger adults, and (3) in the absence of feedback, older adults spontaneously recover marginal knowledge.

## Introduction

Older adults (OAs) typically report a decline in their ability to learn and remember information (e.g., Hertzog and Dixon, 1994). Behavioral data bear out this subjective experience (Balota et al., 2000). Though many aspects of memory decline even in healthy aging, such as explicit memory for specific events (episodic memory), knowledge remains intact and even expands into very old age (Park, 2000). Knowledge includes vocabulary, schemas, facts, and general knowledge about the world. This knowledge influences OAs’ remembering in a variety of ways, sometimes bolstering their accurate remembering and sometimes leading them astray (for a review, see Umanath and Marsh, 2014). OAs also experience more retrieval-related difficulties, reporting more word-finding failures and tip-of-the-tongue states (TOTs; Burke et al., 1991) than do younger adults (YAs). Similar to memory overall, OAs generally perform as well as YAs on metamemory judgments concerning semantic memory or general knowledge (Morson et al., 2015). In contrast, on some episodic tasks, OAs often do not calibrate as accurately as YAs (Souchay et al., 2007), though the literature is mixed (Hertzog and Dunlosky, 2011).

Metacognitive functioning is essential for guiding behavior: Knowing what one knows or does not know enables an individual to determine behaviors. It is also fundamental for learning, in that different efforts, resources, and strategies may be needed based on the basic understanding of whether information is stored in memory or not. That is, what is the cause of this retrieval failure? Is it that I have never learned this and need to now allocate resources to do so, or is it stored but temporarily inaccessible to me? Such metacognition related to the experience of retrieval failures is of particular interest here. Most of the commonly used measures of metamemory do not clearly discriminate between causes of retrieval failure. Specifically, the answer to a question might simply not be stored in memory (i.e., it is unavailable), or it could be stored, but not retrievable at the moment (i.e., it is inaccessible; Tulving and Pearlstone, 1966). Most measures of metamemory tend to ask participants to rate, on a numerical scale, the extent to which they believe they know the answer to a question they cannot answer in the moment. One major example is the “feeling of knowing” measure (Hart, 1965; Koriat, 1993): Participants report how likely they would be to recognize the answer to a question they cannot recall. Such measures have been used extensively and have shown consistent evidence for the strengths and weaknesses in OAs’ memory and metamemory. However, these measures of metacognition do not explicitly determine the “cause” of the failure—the numerical value assigned is assumed to reflect a probability of retrieval, but does not inform the researcher of the underlying phenomenological experience of the participant or of their evaluation as to whether the information is unavailable or inaccessible.

In Coane and Umanath (2019), a novel metamemory tool was developed and tested, capitalizing on natural language use. In that work, OAs’ and YAs’ metacognitive distinction between what is not stored in memory versus merely inaccessible was examined. Knowledge that is merely inaccessible and can be recovered has been termed “marginal knowledge” (Berger et al., 1999). In an initial task, participants answered general knowledge questions in a short-answer format, responding “I don’t know” (DK) or “I don’t remember” (DR) when retrieval failed. When given an opportunity to answer the questions again in a final multiple-choice test, items that had been identified as DR were recognized better than those that had been identified as DK. Critically, OAs and YAs performed quite similarly. These results suggested that DR is associated with a failure in accessibility, whereas DK with a failure in availability. Qualitative analyses of participants’ definitions of what they meant when they used these terms confirmed the underlying phenomenological distinction between *not remembering* and *not knowing*. Indeed, both these empirical and qualitative findings have been replicated with other materials (Umanath et al., 2023; see also Lukasik et al., 2020), providing support for the reliability and validity of participant usage of DR and DK to capture the phenomenological experiences of a lack of accessibility versus availability.

However, one anomalous finding in Coane and Umanath (2019) emerged in their Experiment 3, when the final test was a short-answer task and correct answer feedback had been presented during the initial task. Specifically, OAs ostensibly underestimated their knowledge, as reflected by recovery of a high number of items that they originally claimed they did not know (DK—were not available in memory), far exceeding chance performance. That is, after initially identifying these items as *not known*, OAs answered several of these questions correctly on the final test, more so than did YAs. In fact, final test accuracy for DR and DK items were similar in OAs. There are several possible explanations for such a finding, including but not limited to OAs making a metacognitive error and underestimating the content of their knowledge bases. For example, OAs could be using a potential “face saving” mechanism, whereby admitting lack of knowledge might be less threatening than a retrieval failure (Smith and Clark, 1993), they may have more sophisticated guessing strategies than YAs (particularly when faced with multiple-choice questions; Cyr and Anderson, 2015), they may have more related knowledge with which to integrate new learning from the correct answer feedback (Sitzman et al., 2020), and/or they could experience fluctuations in knowledge accessibility with information coming in and out of accessible range, so to speak. Indeed, with TOTs, OAs are likely to spontaneously recover the answer later, showing “pop-ups” and generally recovering more answers if given more time (Cohen and Faulkner, 1986; Burke et al., 1991). Even across an hour, OAs gained access to more previously non-retrievable general knowledge than did YAs (Umanath, 2016).

Here, through three experiments, we aimed to explore the underlying causes of OAs’ recovery of knowledge that was previously inaccessible—their marginal knowledge. We address two related questions: First, does the overall range of difficulty of the questions matter? Relatively easy questions, like those used in Coane and Umanath (2019), might have led participants, particularly OAs, to rely more on the phenomenological experience of ease of retrieval in making their judgments. Such shifts in comparative evaluation based on phenomenology would be consistent with variability in performance observed in the Remember/Know paradigm typically used in episodic recognition tasks. In that case, performance varies based on what labels (and definitions) are provided to participants (Geraci and McCabe, 2006; Geraci et al., 2009; Williams and Lindsay, 2019). In our previous work, the majority of items were well-known to OAs, potentially resulting in a shift in participants’ phenomenological experiences and skewing their responses. That is, OAs answered over 60% of the questions correctly when they were first presented, leaving few responses to fall in the DR and DK categories. It is possible that OAs in Coane and Umanath (2019) misjudged the contents of their knowledge because of this systematic bias in the selected materials. Specifically, when the majority of items are successfully retrieved and/or perceived as easy, a slightly less familiar item (i.e., more difficult) might be judged as *not remembered* because it cannot be accessed immediately. An item that is even less familiar might be judged as *not known*, not because it is unavailable, but because its accessibility is judged relative to the easy retrieval of other items. Alternatively, the relatively low number of DR and DK responses OAs provided overall (ranging across experiments from 0.11 to 0.16) means that they simply had fewer answers to remember or learn compared to YAs. Therefore, in Experiments 1 and 2, we replicated Coane and Umanath (2019) with normatively difficult questions that were selected to test usability of DR and DK to distinguish accessibility- versus availability-based retrieval failures when retrieval failures are very frequent. Specifically, what happens when all items require more extensive searches through the knowledge base? On the one hand, the difference between what is not accessible (DR) and what is not available (DK) could disappear if the resulting phenomenological experiences are essentially “compressed” due to difficulty. On the other hand, the distinction could also become more salient as the internal comparisons of phenomenology shift away from anchoring on the mental experience of successful retrieval. Simply put, the general knowledge questions used in all studies were more difficult, to extend the search and retrieval space. Thus, this is a strong test of the reliability and generalizability of the DR/DK method.

The second question focused specifically on the role of correct answer feedback in potential new learning and recovery of access to stored general knowledge. For new learning, both basic empirical research and applied research in educational settings highlight the importance of corrective feedback, especially when initial errors are made (Kornell et al., 2009; see Metcalfe, 2017, for a review). The benefits of corrective feedback are especially powerful when the error is semantically related to the correct answer (Kang et al., 2011; Huelser and Metcalfe, 2012). One possibility is that participants attend more to the feedback when it contradicts a response participants thought was correct and therefore encode it more effectively (Potts et al., 2019). This suggests there is a strong episodic component to learning from feedback. According to episodic context accounts of the testing effect (Karpicke et al., 2014), retrieval promotes integration of the specific episodic details, thereby creating a richer memory trace. This richer trace is more resistant to interference (Jacoby and Wahlheim, 2013). In fact, not only do participants retrieve answers better following feedback; they also are more likely to recall specific contextual details of the feedback itself, consistent with an episodic account (Overman et al., 2021). This suggests that one important role of feedback is in updating missing or incorrect knowledge (Metcalfe and Huelser, 2020) and that the ability to encode and retrieve the feedback is critical.

In the context of knowledge-based retrieval failures, feedback can act as an opportunity for new learning or as a cue or reminder of the correct answer, facilitating the recovery of marginal knowledge. Given the importance of episodic contributions to the benefits of feedback and evidence regarding new learning in general (Balota et al., 2000), one might expect that OAs would show a reduced benefit: Deficits in episodic memory should undermine OAs’ ability to acquire and integrate the feedback, especially when the errors are potentially integrated into their knowledge base. For YAs, prior work has shown that feedback can be powerful for stabilizing access to marginal knowledge (Berger et al., 1999). Even a multiple-choice-based retrieval attempt can help recover access for YAs (Cantor et al., 2015). Could the same be true for OAs?

As proposed above, one explanation for OAs’ recovery of marginal knowledge is that they initially underestimated their knowledge bases, making a metacognitive error by labeling items that were actually DR (and inaccessible) but perhaps felt especially difficult to retrieve as DK (and therefore, unavailable). However, it is also possible that after retrieval failures, OAs might have attended more to the feedback provided during the initial phase (Metcalfe et al., 2015). Examination of the response latencies from the final test in Coane and Umanath’s Experiment 3 is consistent with the latter hypothesis: Whereas YAs retrieved correct answers on the final test at the same speed regardless of whether they had responded DR or DK originally, OAs were significantly slower at producing the correct answer for those items given a DK response compared to a DR response, suggesting they were engaging in an effortful search through memory. An alternative is that even if the OAs truly did not have the answers stored in memory, they may have had more related or relevant knowledge in memory, thereby facilitating the acquisition of the new information (Umanath and Marsh, 2014). To address these questions, in a final experiment, we replicated the short-answer test version of the study with online samples and manipulated the presence of correct answer feedback. Importantly, systematically manipulating the presence of feedback allows us to examine the extent to which OAs are misjudging the availability of information in memory. Items that are truly *not known* would not be expected to be answered correctly, unless feedback is provided and participants learn from that feedback. If OAs demonstrate such a distinction in correct responses on the final test for DR versus DK items when no feedback is provided with more DR items being answered correctly than DK ones, it would provide evidence against the hypothesis that OAs are simply making metacognitive errors.

In sum, in the experiments reported here, we examined whether participants are accurate in determining whether an item is inaccessible (*not remembered*) or unavailable (*not known*) when retrieval failures are quite frequent. As in previous work (Coane and Umanath, 2019; Umanath et al., 2023), we expected final test accuracy to be higher for items initially judged as *not remembered* than those judged as *not known* for both age groups. When the final test is a multiple-choice format, a temporarily inaccessible item should be correctly recognized more often than an item that is not part of the knowledge base. When the final test requires effortful retrieval (i.e., a short-answer test) and feedback is given, the feedback should serve as a “reminder” and be subsequently retrieved at a greater rate for marginal knowledge (*not remembered*) than when the feedback acts as a new learning opportunity (when the item was deemed *not known*), perhaps especially for OAs who routinely show deficits in episodic learning. But particularly, in the absence of feedback, items *not remembered* should be more likely to be correctly answered, due to spontaneous retrieval or continued search in memory, compared to items *not known*. This is the key question we addressed in the final experiment.

## Experiment 1

Experiments 1 and 2 served as replications and extensions of prior work (Coane and Umanath, 2019) to test participants’ abilities to distinguish failures in accessibility from failures in availability (Tulving & Pearlstone, 196) when items are unfamiliar or obscure (i.e., when retrieval is less likely to succeed and when the difference between these causes of failures could be less apparent). In Experiment 1, participants were not given correct answer feedback after initial exposure to short-answer general knowledge questions and were administered a final multiple-choice recognition test.

### Method

#### Participants

In Exp. 1, 56 YAs (35 women) participated via participant pools at Claremont McKenna and Colby Colleges, earning course credit or $10 for their participation, and 33 community-dwelling OAs (27 women) from both surrounding communities participated for $10/h. Sample size was determined based on the effect size for OAs (who had a smaller effect size) for the difference in accuracy between initial DR and DK responses on the final test in Experiment 2 in Coane and Umanath (2019) and estimated power of 0.9. The minimum sample was 27 in each age group; YAs were over-sampled because both labs were recruiting and testing simultaneously. All but one of the OAs also completed the Mini Mental State Exam (MMSE; Folstein et al., 1975). OAs scored higher than YAs in vocabulary (Shipley, 1940), *t*(74) = 6.75, *p* < 0.001, *d* = 1.48, and had more years of education, *t*(32.07) = 11.95, *p* < 0.001, *d* = 2.62. See Table 1 for full demographic information.

**Table 1:** Participant demographic information for experiments 1, 2, and 3.

	Age	Education	Shipley	MMSE
Experiment 1				
YAs (*N* = 56; 35 women)	*M* = 18.89 SD = 1.00 Range = 17–21	*M* = 12.68 SD = 0.83 Range = 12–15	*M* = 31.16 SD = 2.88	
OAs (*N* = 33; 27 women)	*M* = 74.79 SD = 6.17 Range = 62–88	*M* = 16.45 SD = 2.11 Range = 12–21	*M* = 35.27 SD = 2.59	*M* = 28.75 SD = 1.08
Experiment 2				
YAs (*N* = 55; 39 women)	*M* = 19.09 SD = 1.11 Range = 17–22	*M* = 13.05 SD = 1.03 Range = 12–15	*M* = 30.89 SD = 3.57	
OAs (*N* = 32; 23 women)	*M* = 73.81 SD = 8.01 Range = 62–88	*M* = 16.86 SD = 2.43 Range = 12–23	*M* = 36.50 SD = 2.95	*M* = 28.56 SD = 1.37
Experiment 3				
Feedback				
YAs (*N* = 44; 21 women)	*M* = 21.80 SD = 1.76 Range = 18–24	*M* = 14.25 SD = 2.15 Range = 8–18	*M* = 31.11 SD = 3.94	
OAs (*N* = 43; 31 women)	*M* = 64.47 SD = 3.59 Range = 60–74	*M* = 15.45 SD = 2.56 Range = 12–20	*M* = 36.77 SD = 2.20	
No feedback				
YAs (*N* = 43; 19 women)	*M* = 21.91 SD = 1.93 Range = 18–24	*M* = 13.95 SD = 1.70 Range = 12–17	*M* = 30.86 SD = 3.79	
OAs (*N* = 47; 25 women)	*M* = 66.89 SD = 6.05 Range = 60–93	*M* = 14.94 SD = 2.38 Range = 10–23	*M* = 35.68 SD = 2.96	

#### Materials

To elicit a high rate of “don’t remember” (DR) and “don’t know” (DK) responses from both YAs and OAs, 70 difficult general knowledge questions (GKQs) from Tauber et al. (2013) were selected on the basis of particularly low reported retrieval rates in YAs (*M* = 0.26, SD = 0.14, range = 0.00–0.58). Based on norming data from OAs (Coane and Umanath, 2021), multiple-choice (MC) accuracy for these questions ranged from 0.10–0.98 (*M* = 0.53). Thirty additional GKQs from Tauber et al. with high retrieval rates were used as filler items (*M* = 0.76, SD = 0.08, range = 0.51–0.93). The questions had simple one- or two-word answers, (e.g., *What is the last name of the author who wrote “Our Town”?*, answer: *Wilder*). As a filler task, participants were provided a packet that contained simple arithmetic problems and Sudoku puzzles.

#### Procedure

The experiment was conducted using E-Prime software (Schneider et al., 2012), with YAs tested in the lab and OAs tested both in the lab and at a local senior college, all tested individually. Participants answered 100 GKQs in a short-answer format after being provided an example and told that some may be difficult. They were asked to respond “don’t know” (DK) or “don’t remember” (DR) if they were unsure of an answer. Critically, participants were not provided with explicit instructions on the difference between these two responses but simply told to use their best judgment (see Coane and Umanath, 2019). Questions were presented individually in random order. The task was self-paced, and participants typed their response directly into the computer. After a 5-min filled delay (participants could freely choose to do arithmetic or Sudoku), participants completed a multiple-choice (MC) test of the same GKQs in a different random order with five possible responses: The correct response and four plausible alternatives. The position of the correct answer varied across all five options an equal number of times across all questions. Participants then answered two open-ended questions about their use of DK and DR in a randomized order. Specifically, they were asked “What did you mean when you used “I don’t know/I don’t remember” in the first part of the study?.” Results from these questions are not reported here; they were examined to ensure that participants discriminated between the two options, which most participants did. Finally, all participants completed the Shipley (1940) vocabulary task, and OAs also completed the MMSE (Folstein et al., 1975).

### Results and discussion

The analyses below, and in subsequent experiments, only include responses to the difficult questions; analyses on fillers are not reported. Where relevant, we applied a Bonferroni correction for multiple comparisons, and in cases of violations of assumptions of sphericity or normality, corrected degrees of freedom are reported (Greenhouse–Geisser for ANOVAs). For effect sizes, we report partial *η*^2^ for ANOVAs and Cohen’s *d* for t*-*tests.

#### Initial short-answer performance

All responses were coded as incorrect (including both errors of omission and commission), correct (including minor spelling errors or morphological variations), DR, or DK. Errors of omission only occurred on nine trials (accounting for 0.001 of all trials). Omission errors were made by three YAs and three OAs; all these participants provided similar rates of correct, incorrect, DR, and DK responses to the participants who did not make any omission errors all *t*s ≤ 1.66, *p*s ≥ 0.101. Given the lack of independence in the responses (i.e., a higher rate of correct responses would necessarily result in fewer DR or DK responses), we report a series of independent samples *t*-tests comparing response proportions as a function of age (*cf.* Umanath et al., 2023). See Table 2 for means.

**Table 2:** Participant performance on exposure phase of Experiments 1, 2, and 3 (standard errors in parentheses).

	Correct	DR	DK	Incorrect
Experiment 1				
Younger adults	0.06 (0.03)	0.16 (0.01)	0.65 (0.03)	0.13 (0.01)
Older adults	0.23 (0.03)	0.19 (0.02)	0.34 (0.02)	0.24 (0.01)
Experiment 2				
Younger adults	0.06 (0.01)	0.13 (0.01)	0.65 (0.02)	0.17 (0.02)
Older adults	0.24 (0.03)	*0.*20 (0.02)	0.34 (0.03)	0.22 (0.02)
Experiment 3				
Feedback				
Younger adults	0.06 (0.02)	0.09 (0.01)	0.73 (0.03)	0.13 (0.02)
Older adults	0.25 (0.02)	0.12 (0.02)	0.43 (0.03)	0.19 (0.02)
No feedback				
Younger adults	0.09 (0.01)	0.11 (0.02)	0.71 (0.03)	0.09 (0.02)
Older adults	0.33 (0.02)	0.17 (0.02)	0.33 (0.03)	0.17 (0.02)

The overall low accuracy rate does confirm that the items were quite difficult. OAs answered more questions correctly than YAs, *t*(38.39) = 5.89, *p* < 0.001, *d* = 1.56. They were also more likely than YAs to respond incorrectly, *t*(87) = 5.64, *p* < 0.001, *d* = 1.24. In contrast, YAs responded DK more often than OAs, *t*(87) = −8.89, *p* < 0.001, *d* = −1.95. The use of DR did not differ across age groups, *t*(87) = 1.10, *p* = 0.273, *d* = 0.24.

#### Final MC test performance

**Figure 1:**
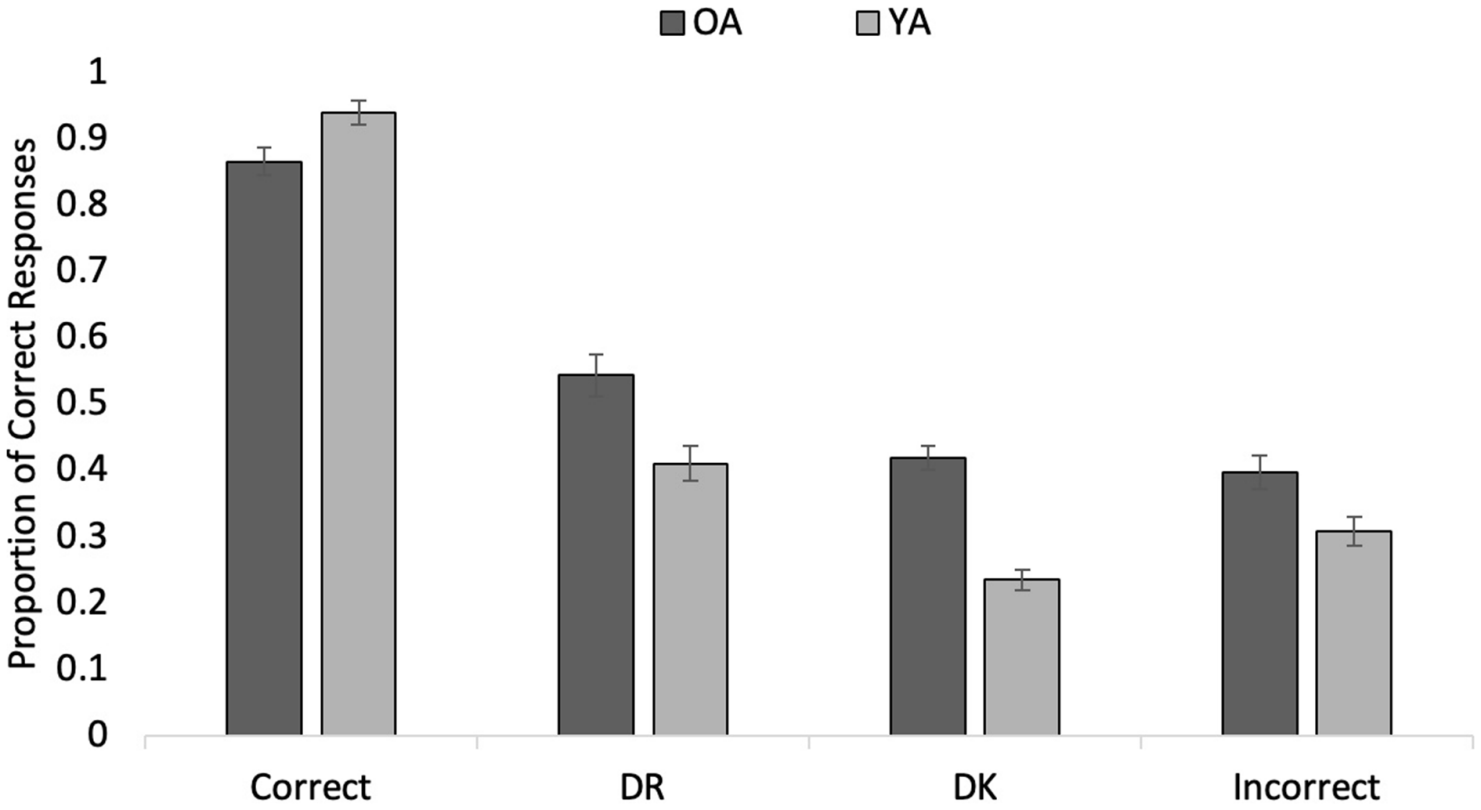
Older and younger participants’ Experiment 1 accuracy on the final multiple-choice test as a function of initial response. Error bars represent standard error of the mean.

A 4 (Response) × 2 (Age) ANOVA was conducted to examine the proportion of correct selection on the final MC test as a function of the initial test response. Data from 30 OAs and 44 YAs were included due to empty cells (i.e., some participants did not have data for the final test because they never provided one or more of the response options during the exposure phase). As seen in Figure 1, OAs answered more questions correctly (*M* = 0.56, SE = 0.01) than YAs (*M* = 0.47, SE = 0.01), *F*(1, 72) = 26.63, *p* < 0.001, *η*_p_^2^ = 0.27. Importantly, MC accuracy significantly varied as a function of participants’ initial test responses, *F*(2.47, 178.38) = 271.38, *p* < 0.001, *η*_p_^2^ = 0.79. Participants were able to maintain correct responses, recognizing the vast majority of final questions correctly if they were able to generate the correct answer initially (*M* = 0.90, SE = 0.02). Initial DR response items had the next highest mean selection accuracy rate (*M* = 0.48, *SE* = 0.02), followed by initial DK responses (*M* = 0.33, SE = 0.01) and by initially incorrect responses (*M* = 0.35, SE = 0.02). All pairwise comparisons were significant (*p*s < 0.001, all *d*s ≥ 0.71), other than the difference between initial DK and incorrect responses (*p* > 0.999, *d* = 0.11). Thus, our results provide evidence that both age groups successfully discriminated between DK and DR responses, replicating Coane and Umanath (2019: Exp. 2) when material was much more difficult and the distribution of phenomenology shifted away from mainly retrieval success.

The analyses further revealed a significant Age by Response interaction, *F*(2.47, 178.38) = 11.81, *p* < 0.001, *η*_p_^2^ = 0.14 (see Figure 1). Tests of simple effects indicated that, in both age groups, the main effect of Response was significant: For OAs, *F*(3, 70) = 98.78, *p* < 0.001, *η*_p_^2^ = 0.81, and for YAs, *F*(3, 70) = 327.09, *p* < 0.001, *η*_p_^2^ = 0.93. The interaction was driven by the fact that, whereas for YAs, all pairwise comparisons were significant (all *p*s ≤ 0.043, all *d*s > 0.30), OAs’ final test accuracy for initial DK and Incorrect items did not differ (*p* > 0.999, *d* = 0.20), whereas all other pairwise comparisons were significant (all *p*s ≤ 0.010, all *d*s > 0.69). Importantly, for both age groups, items initially *not remembered* were correctly recognized more often than those originally *not known* (both *p*s ≤ 0.005, *d*_YA_ = 0.69 and *d*_OA_ = 0.76). The lack of a difference for OAs between DK and Incorrect items might reflect a true lack of knowledge (i.e., an initial guess led to an incorrect response – but the lack of prior knowledge did not indicate an ability to recognize the correct answer among foils). These results confirm those of Coane and Umanath (2019) and extend the differences in self-reported inaccessibility and unavailability to a new set of very difficult questions.

## Experiment 2

In Experiment 2, after attempting to answer the short-answer GKQs initially, participants were given correct answer feedback regardless of their response. In the case of temporary retrieval failures or errors, the feedback should serve as a reminder and facilitate subsequent retrieval. In contrast, if the information is truly not known (is unavailable), the feedback should act as a new learning episode. As discussed above, using difficult GKQs may facilitate separation of initial DR versus DK items, which was not observed in Coane and Umanath (2019: Exp. 3) using easier questions. By modifying the materials in this way, we might increase the salience of information that is not available vs. not accessible, reducing the number of DK responses that refer to very difficult to access but ultimately available knowledge. Furthermore, for very obscure knowledge, the feedback would be more likely to be the first time individuals are exposed to this information. It is also possible that obscure knowledge has fewer connections to prior knowledge, which could make the feedback less effective because OAs would not be able to capitalize on their ability to integrate new learning (Metcalfe, 2017).

### Method

#### Participants

Fifty-five YAs (39 women) and 33 OAs (23 women) were tested. One OA participant’s data were excluded because of a score of 24 on the MMSE, which is the standard cut-off. As in Experiment 1, OAs outperformed YAs on the vocabulary task, *t*(85) = 7.51, *p* < 0.001, *d* = 1.67, and had more years of education, *t*(37.51) = 8.42, *p* < 0.001, *d* = 2.26. See Table 1 for demographic information.

#### Materials and procedure

The same GKQs from Experiment 1 were used. In the initial short-answer task, after a retrieval attempt, participants were shown the correct answer for 2 s. The final test was another short-answer test, without correct answer feedback provided.

### Results and discussion

#### Initial short-answer performance

Participant responses were coded as in Experiment 1. Errors of omission occurred on 41 trials (less than 1% of all trials). One YA committed 26 omission errors; the rest were made by four YAs (who committed between 1 and 3 errors each) and three OAs (who each made one omission error). These participants did not differ in terms of the proportion of correct, DR, DK, and incorrect responses from the participants who never made omission errors (all *t*s ≤ 1.51, *p*s ≥ 0.134). A series of *t*-tests compared response proportions as a function of age (see Table 2). OAs correctly answered more questions than YAs, *t*(33.78) = 6.12, *p* < 0.001, *d* = 1.72. OAs also used DR more than YAs, *t*(85) = 2.77, *p* = 0.007, *d* = 0.62, and were more likely to generate an error response, *t*(85) = 2.03, *p* = 0.045, *d* = 0.45. In contrast, YAs used DK more often than OAs, *t*(85) = −8.49, *p* < 0.001, *d* = −1.89. Overall, other than the difference in use of DR between Experiments 1 and 2 (although the numerical trend in Experiment 1 was similar), the distribution of responses was similar. It was also similar to our earlier work (Coane and Umanath, 2019) in terms of ordering of responses across ages.

#### Final short-answer test performance

Errors of omission only occurred on 37 trials (less than 1% of trials; 30 were made by the same YA who made a large number of such errors during the exposure phase). The same 4 (Response) × 2 (Age) ANOVA was used to analyze performance on the final short-answer test. Data from 30 OAs and 46 YAs were included due to empty cells. These analyses revealed a main effect of Age; OAs (*M* = 0.64, SE = 0.02) again outperformed YAs [*M* = 0.53, SE = 0.02, *F*(1, 74) = 10.81, *p* = 0.002, *η*_p_^2^ = 0.13]. Accuracy on the final test differed as a function of initial test phase response, *F*(2.48, 183.21) = 194.61, *p* < 0.001, *η*_p_^2^ = 0.73. Correct responses were almost always maintained (*M* = 0.96, SE = 0.02). Initial Incorrect responses (*M* = 0.56, SE = 0.03) had the next highest accuracy rate, followed by DR responses (*M* = 0.51, SE = 0.03), and DK responses (*M* = 0.31, SE = 0.02). All pairwise comparisons were significant (*p*s < 0.001, *d*s ≥ 0.99), other than the difference between DR and Incorrect responses (*p* = 0.444, *d* = 0.18). Thus, across age groups, participants were more likely to answer a question correctly that they initially could *not remember*, relative to questions for which they claimed they did not initially know the answer. This occurred despite receiving correct answer feedback for all questions, suggestive that having a DR experience (i.e., one of inaccessibility) and receiving feedback facilitated recovery of marginal knowledge. It is worth noting that, in contrast to Experiment 1, the feedback resulted in correction of incorrect responses, such that initially Incorrect responses were similar in performance to initially *not remembered* responses. In addition, interestingly, the short-answer format of this test did not result in reduced performance for OAs as might be expected for switching from recognition to recall.

**Figure 2:**
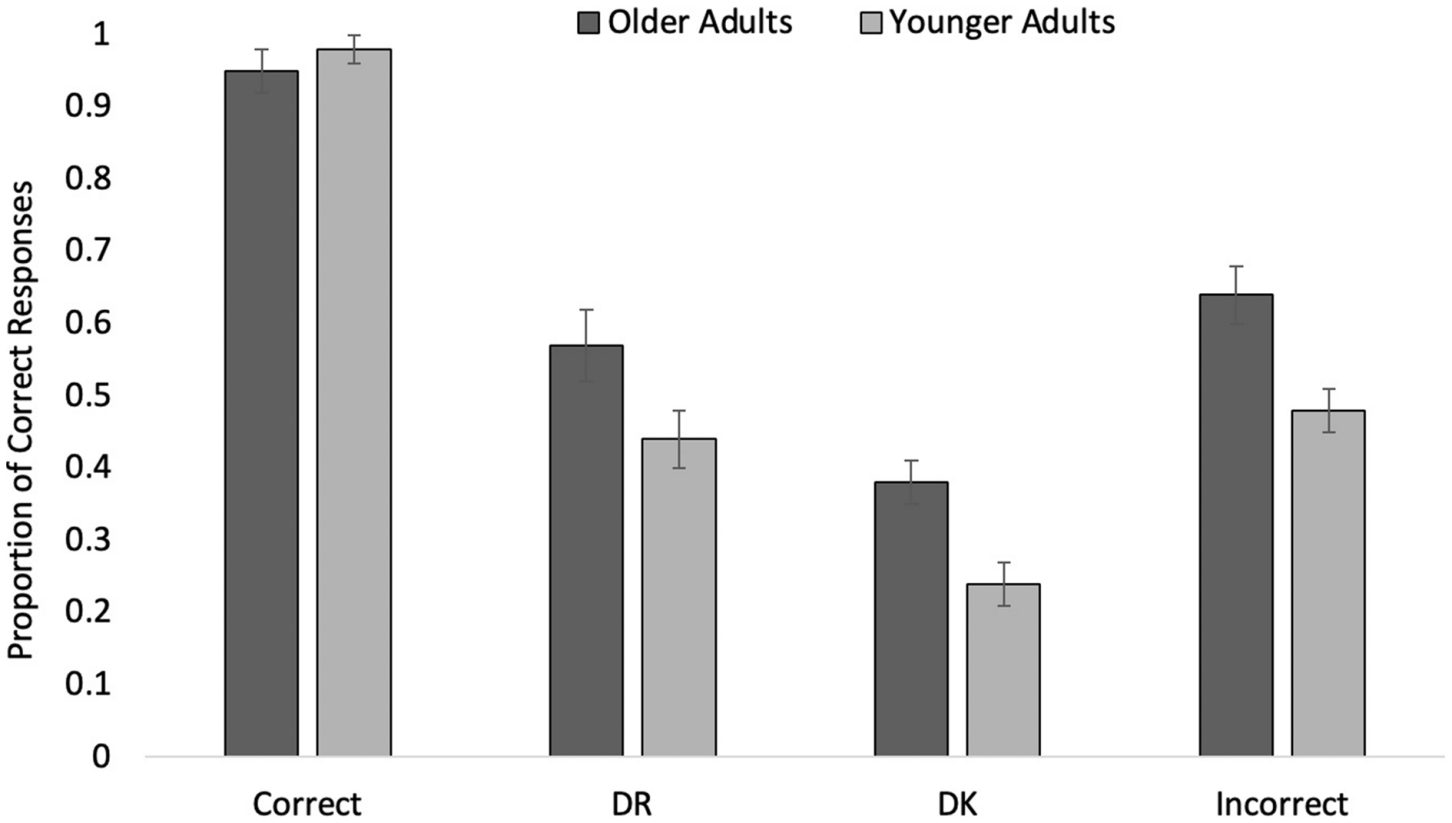
Older and younger participants’ Experiment 2 shorts-answer final test performance as a function of initial short-answer response. Error bars represent standard error of the mean.

Age and Response interacted, *F*(2.48, 183.21) = 5.08, *p* = 0.002, *η*_p_^2^ = 0.06 (see Figure 2). Both OAs’ and YAs’ accuracy on the final test as a function of initial test response followed the same pattern of results, as described above, *F*(3, 72) = 100.83, *p* < 0.001, *η*_p_^2^ = 0.81 and *F*(3, 72) = 246.36, *p* < 0.001, *η*_p_^2^ = 0.91, respectively. In both age groups, all pairwise comparisons were significant (all *p*s ≤ 0.001, *d*s ≥ 0.89) other than the difference between DR and Incorrect (both *p*s ≥ 0.941, *d*s ≤ 0.39). The interaction, as can be seen in the figure, is largely due to the fact that, relative to initially Correct items, YAs showed larger decreases in accuracy (ranging from 0.50 to 0.74) for the other three item categories than OAs (differences ranging from 0.30 to 0.57). Importantly, both age groups showed a robust performance advantage for DR items than for DK items (*M*_OA_ = 0.20, SE = 0.04, *d* = 1.27; *M*_YA_ = 0.20, SE = 0.03, *d* = 0.89), suggesting that the lack of an effect in Coane and Umanath (2019) might have been due to overall item ease and frequency of retrieval success altering OAs’ assessments of availability.

Furthermore, as can be seen in Figure 2, OAs outperformed YAs on the final test in all categories other than Correct (*p* = 0.342, *d* = 0.28). That OAs were more accurate than YAs on initial DR items (*p* = 0.022, *d* = 0.55) indicates greater recovery of marginal knowledge than YAs. That they were more accurate on initial Incorrect items (*p* = 0.002, *d* = 0.82) means that they were better able to correct their mistakes than YAs (*cf.* Sitzman et al., 2020). That they were more accurate on the DK items (*p* < 0.001, *d* = 0.94) suggests they might have particularly benefited from the correct answer feedback. Such findings are exciting in that they demonstrate that OAs can recover marginal knowledge, correct their errors, and potentially even learn something new from a single trial with feedback, and maintain the information at least for a relatively brief delay. As previously demonstrated by Sitzman et al. (2020), OAs were able to correct their errors. Here, we find evidence that OAs could also be acquiring episodically-encoded new knowledge better than YAs. However, examination of the means for initial DK items reveals that OAs’ final test performance was still quite high, and significantly higher than that of YAs. This raises the concern that participants, OAs in particular, are making a metacognitive error. We address this in the next experiment.

## Experiment 3

In Experiment 2, correct answer feedback was included after the initial short-answer test. We hypothesized that the feedback would be more beneficial for *not remembered* items for which it would act as a reminder than for *not known* items for which participants would presumably need to learn something new (Metcalfe, 2017; Sitzman et al., 2020). This hypothesis was confirmed. Given the data regarding OAs’ challenges in learning new information (Balota et al., 2000), this is surprising and again raises the question of whether OAs are misusing DK in some cases, perhaps reflecting a metacognitive error on their part. Specifically, an item that is particularly difficult to access might be erroneously judged as unavailable. Fluctuations in knowledge access (Umanath, 2016) could then allow this information to be recovered. Alternatively or in conjunction, OAs might be more attentive to and encode the feedback more efficiently (Metcalfe et al., 2015), their larger knowledge base might facilitate retention of the newly learned information (Cyr and Anderson, 2012) or they may spontaneously recover access to DR items over the course of the study (Umanath, 2016). The presence of correct answer feedback in the previous studies masks the ability to clearly discriminate between these various possibilities.

To more closely examine these explanations, we conducted a final experiment in which we manipulated the presence of the correct answer feedback. A no-feedback condition allows us to create a situation in which participants receive no external cues or other information (e.g., lures in a multiple-choice questions) to help jog their memory and regain, or gain for the first time, the answers. If participants are correct in their metacognitive judgment of DK items – that they refer to information that is not available in the participants’ memory – we would predict floor or close to floor performance on the final test. If OAs still outperform YAs for DR items on the final test even when feedback is not provided, this would suggest some recovery of marginal knowledge (although we acknowledge this does not rule out the alternative explanation of feedback processing). Again, critically, if their assessment of inaccessibility vs. unavailability is accurate, no such recovery should occur for DK items. Therefore, when retrieval fails (i.e., when a DR or DK response is given) and no feedback is provided, any correct answer on the final test would be due to spontaneous recovery: This is what we tested in the final experiment. A condition with Feedback allowed us to replicate the results of Experiment 2 in an online sample.

### Method

#### Participants

To determine sample size, we conducted an *a priori* test in G*Power (Faul et al., 2007) using the effect size of 0.06 for the Age by Response interaction obtained in the final open-ended response task in Experiment 2. We estimated four groups (created by the crossing of Age and Feedback condition) and four measurements (the four response options). Assuming a correlation between repeated measures of 0.23 (this was the average of the correlations observed in Experiment 2), a total sample of 76 would provide power to detect an effect of 0.96. We increased the target sample to approximately 40 per age group because of the addition of a third factor (feedback) and to account for the potentially greater variability in online populations and higher attrition rates. The presence of feedback was manipulated between-subjects because we wanted to ensure that enough DR and DK responses occurred in the initial phase for the conditional analyses to be appropriate and meaningful.

A total of 96 participants started the Feedback condition and 106 started the No Feedback condition. In the former, eight timed out, and in the latter, seven timed out; these data were not included in the analyses. In the Feedback condition, 43 OAs (31 women) and 44 YAs (21 women, 2 other) completed the study. Based on responses to the screening questions (see below), data from one YA were omitted from analyses.

In the No Feedback condition, 51 OAs and 45 YAs completed the study. An additional seven participants were excluded from analyses due to an error when the study was posted to Prolific (they had already completed the Feedback condition). After screening participants based on their responses to the integrity questions (see below), data from 47 OAs (25 women) and 43 YAs (19 women, 3 other) were included in the final analyses (see Table 1 for demographic information).

OAs had more years of education, *t*(166.27) = 3.23, *p* = 0.001, *d* = 0.49, and scored higher on Shipley, *t*(152.79) = 10.44, *p* < 0.001, *d* = 1.58 compared to YAs. The study took approximately 50 min to complete. Consistent with the guidelines of Prolific,^1^ older participants received $13.64/h for their time and younger participants received $14.31/h (although the pay rate was the same, OAs took slightly longer than YAs).

#### Materials and procedure

The experiments were programmed on Gorilla Experiment Builder^2^ (Anwyl-Irvine et al., 2020) and were conducted on Prolific.^3^ The stimuli and procedure were mostly identical to those used in Experiment 2. The main differences were that, during the exposure phase, participants could type in a response or click on one of two buttons marked “I don’t remember” and “I don’t know.” In the Feedback condition, after entering a response or selecting one of the two buttons, the correct answer was presented for 2 s. In the No Feedback condition, participants simply advanced to the next screen. In addition, to account for the online administration, three additional questions were added at the end of the study: Participants were asked whether they had looked up answers online, whether they had completed the study in more than one sitting, and if their data should be excluded for any reason. Finally, participants provided demographic information and read a debriefing statement, after which they received their monetary compensation directly from Prolific.

### Results and discussion

#### Screening questions

In the Feedback condition, all participants confirmed having completed the study in a single setting and that they had not looked up the answers online. One younger adult requested their data be excluded but did not provide a reason other than poor performance (their data were excluded, as per their request). In the No Feedback condition, one OA and one YA reported they did not complete the task in a single setting, one YA admitted to looking up answers online, and three OAs reported their data should be excluded because of participation in a similar study.

#### Initial short-answer performance

As in the previous studies, all open-ended responses were coded as correct or incorrect. On some trials, participants entered a response and clicked the DR or DK button (these occurred fewer than 20 times). When this occurred, if the response they provided was correct, we kept the correct answer; otherwise, it was coded as DR or DK. Other than one older participant in the Feedback condition who did not provide a response on 43 trials (61%), only nine of the remaining responses were omission errors (four in the No Feedback condition and five in the Feedback condition). All omission errors in the No Feedback condition were made by four different OAs and, in the Feedback condition, one YA omitted two responses, two other YAs omitted one response each, and one OA omitted one response. Overall, omission errors accounted for less than 1% of responses in both conditions.

The proportion of each response as a function of age was examined in a series of 2 (Condition) × 2 (Age) ANOVAs. See Table 1 for means. OAs (*M* = 0.29, SE = 0.02) provided more correct answers than YAs (*M* = 0.08, SE = 0.02), *F*(1, 173) = 92.48, *p* < 0.001, *η*_p_^2^ = 0.35. Participants in the No Feedback condition (*M* = 0.21, SE = 0.02) answered more questions correctly than those in the Feedback condition (*M* = 0.16, SE = 0.02), *F*(1, 173) = 6.45, *p* = 0.012, *η*_p_^2^ = 0.04. It is possible that knowing feedback would be provided had a small effect on participants’ motivation or willingness to guess. The interaction was not significant, *F*(1, 173) = 1.25, *p* = 0.266, *η*_p_^2^ = 0.007.

OAs (*M* = 0.15, SE = 0.01) also were more likely to respond with DR than YAs (*M* = 0.10, SE = 0.01), *F*(1, 173) = 11.08, *p* = 0.001, *η*_p_^2^ = 0.06 and participants in the Feedback condition (*M* = 0.10, SE = 0.01) used DR less than those in the No Feedback condition (*M* = 0.14, SE = 0.01), *F*(1, 173) = 5.64, *p* = 0.019, *η*_p_^2^ = 0.03. The interaction was not significant, *F* < 1.0, *p* = 0.356, *η*_p_^2^ = 0.005.

Turning to DK responses, OAs (*M* = 0.38, SE = 0.02) were less likely to respond DK than YAs (*M* = 0.72, SE = 0.02), *F*(1, 173) = 135.28, *p* < 0.001, *η*_p_^2^ = 0.44. The effect of Condition approached significance, *F*(1, 173) = 3.56, *p* = 0.061, *η*_p_^2^ = 0.02: Participants in the Feedback group (*M* = 0.58, SE = 0.02) used DK slightly more than those in the No Feedback group (*M* = 0.52, SE = 0.02). The interaction was not significant, *F*(1, 173) = 2.31, *p* = 0.129, *η*_p_^2^ = 0.01.

Finally, Incorrect responses were given more frequently by OAs (*M* = 0.18, SE = 0.01) than by YAs (*M* = 0.11, SE = 0.01), *F*(1, 173) = 16.53, *p* < 0.001, *η*_p_^2^ = 0.09. There was a trend in the data that participants in the Feedback condition (*M* = 0.16, *SE* = 0.01) were more likely to make errors than those in the No Feedback condition (*M* = 0.13, SE = 0.01), *F*(1, 173) = 3.18, *p* = 0.076, *η*_p_^2^ = 0.02. There was no interaction, *F*(1, 173) = 0.44, *p* = 0.506, *η*_p_^2^ = 0.003.

In sum, although the two conditions differed slightly in the distribution of responses, the overall pattern was similar: DK responses were the most common response, especially for YAs. OAs were able to correctly answer the questions more than YAs and also made more errors and used DR more often. It appears as if the participants in the Feedback condition seemed to be more willing than those in the No Feedback condition to provide a response, as evidenced by the higher rates of Correct and Incorrect responses. It is possible that knowing feedback would be provided encouraged participants to guess.

#### Final short-answer test performance

**Figure 3:**
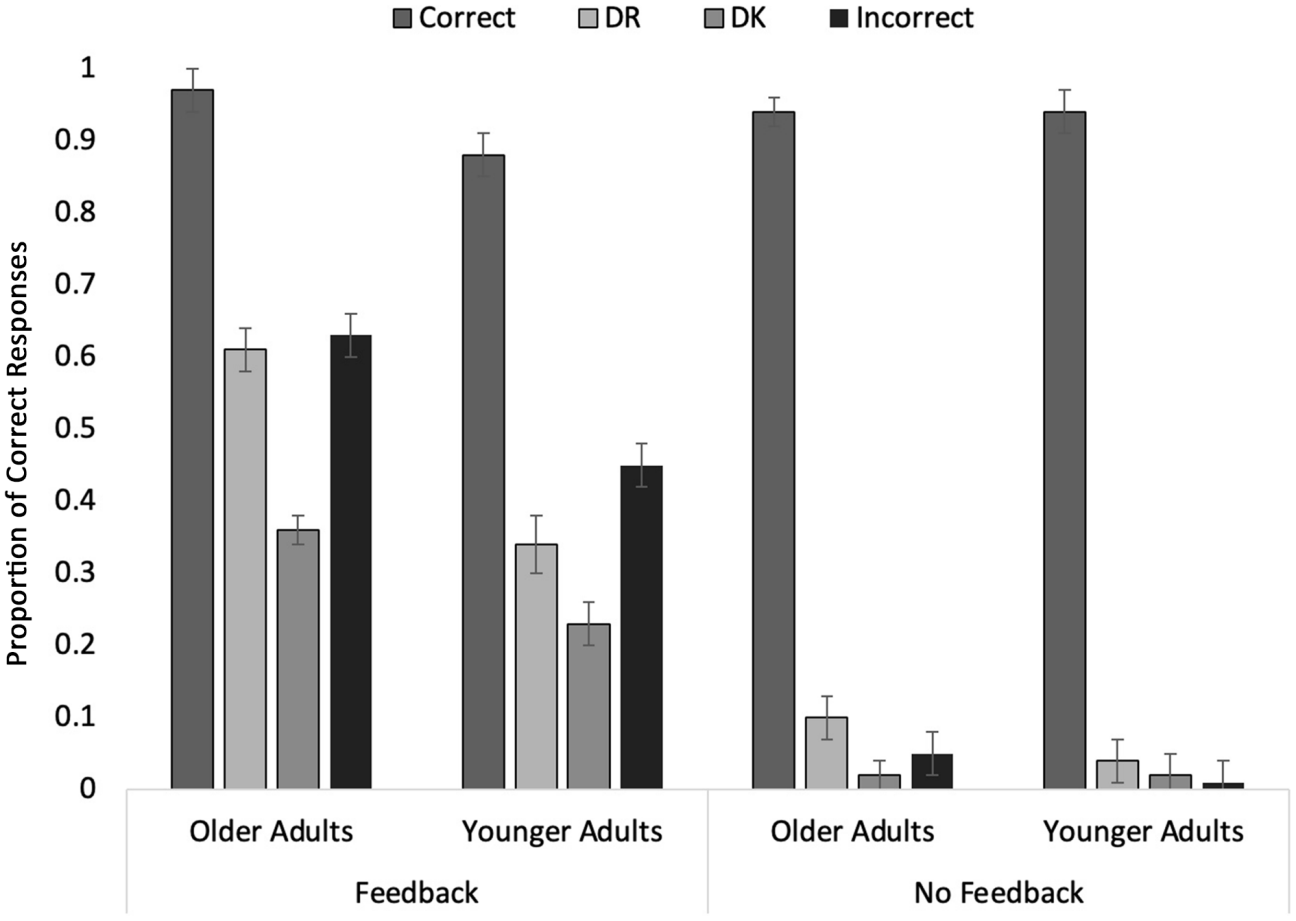
Older and younger participants’ Experiment 3 final test performance as a function of condition and initial response. Error bars represent standard error of the mean.

The proportion of correct responses as a function of the response provided during the initial short-answer task and feedback during the initial short-answer task was analyzed in a 2 (Age) × 4 (Response) × 2 (Condition) mixed ANOVA. Data from 75 OAs (35 in the Feedback condition and 40 in the No Feedback condition) and 59 YAs (27 in the Feedback condition and 32 in the No Feedback condition) were included in the analyses. The data are depicted in Figure 3.

As in the initial short-answer task, OAs (*M* = 0.46, SE = 0.01) correctly answered more questions than YAs (*M* = 0.36, SE = 0.01), *F*(1, 130 = 25.90, *p* < 0.001, *η*_p_^2^ = 0.17). Participants who received feedback (*M* = 0.56, SE = 0.01) answered more questions correctly than those who did not receive feedback (*M* = 0.26, SE = 0.01), *F*(1, 130) = 232.02, *p* < 0.001, *η*_p_^2^ = 0.64. The initial response also significantly affected final test accuracy, *F*(2.74, 355.90) = 768.62, *p* < 0.001, *η*_p_^2^ = 0.86: Initially correct answers were almost always maintained (*M* = 0.93, SE = 0.01); Incorrect (*M* = 0.28, SE = 0.02) and DR (*M* = 0.27, SE = 0.02) items were answered correctly at similar rates; and DK items were the most poorly recalled (*M* = 0.16, SE = 0.01). All pairwise comparisons were significant (all *p*s ≤ 0.001) other than the comparison between Incorrect and DR (*p* ≥ 0.999).

All three 2-way interactions were significant (see Figure 3). The Age by Condition interaction, *F*(1, 130) = 3.73, *p* < 0.001, *η*_p_^2^ = 0.10, revealed that, in terms of overall accuracy, OAs (*M* = 0.64, SE = 0.02) outperformed YAs (*M* = 0.47, SE = 0.02) in the Feedback condition, *F*(1, 130) = 35.92, *p* < 0.001, *η*_p_^2^ = 0.22. However, there was no difference between OAs (*M* = 0.28, SE = 0.02) and YAs (*M* = 0.25, SE = 0.02), in the No Feedback condition, *F*(1, 130) = 1.04, *p* = 0.310, *η*_p_^2^ = 0.008. This suggests that OAs made better use of the feedback than did the YAs.

The Age by Response interaction, *F*(2.74, 355.90) = 4.52, *p* = 0.005, *η*_p_^2^ = 0.03, revealed that although both age groups showed a similar ordering, there were some important differences. Specifically, for the OAs (all *p*s ≤ 0.001), *F*(3, 128) = 366.26, *p* < 0.001, *η*_p_^2^ = 0.90, all pairwise comparisons were significant (*p*s < 0.001) other than that between DR and Incorrect (*p* > 0.999). For YAs, *F*(3, 128) = 317.12, *p* < 0.001, *η*_p_^2^ = 0.88, similarly, final test performance did not differ for DR and Incorrect items (*p* = 0.627), but accuracy between DR and DK items did not differ either (*p* = 0.103). All other pairwise comparisons were significant (*p*s < 0.001). Thus, although both age groups did retain correct responses, OAs’ final performance for initial retrieval failures and errors appeared to be more sensitive to variations in accessibility than YAs’.

The Condition by Response interaction, *F*(2.74, 355.90) = 81.99, *p* < 0.001, *η*_p_^2^ = 0.39, indicated that, when feedback was given, correct responses were highest following initial Correct responses (*M* = 0.92, SE = 0.02), then initial Incorrect responses (*M* = 0.54, SE = 0.02), then DR responses (*M* = 0.47, SE = 0.02), and finally DK responses (*M* = 0.30, SE = 0.02), *F*(3, 128) = 211.68, *p* < 0.001, *η*_p_^2^ = 0.83. All pairwise comparisons were significant (all *p*s ≤ 0.001, *d*s ≥ 0.70) other than the difference between Incorrect and DR, which approached significance (*p* = 0.093, *d* = 0.20). However, in the absence of Feedback, whereas initial correct responses (*M* = 0.94, SE = 0.02) were well-maintained, correct responses following DR (*M* = 0.07, SE = 0.02), Incorrect (*M* = 0.03, SE = 0.02), and DK (*M* = 0.02, SE = 0.02) responses were low and did not differ from one another (all *p*s ≥ 0.272), *F*(3, 128) = 553.19, *p* < 0.001, *η*_p_^2^ = 0.93. That participants perform at or near floor suggests that in this condition, they are accurate about deeming these items DK during the initial phase. Any correct DR items, we would argue represent spontaneous recovery of marginal knowledge. Given the short delays included in the study, participants did not have a lot of time to mull over the questions and potentially recover answers. We note that, in this analysis, the difference between DR and DK was not significant, a finding we address below.

The three-way interaction was not significant, *F*(2.74, 355.90) = 1.03, *p* = 0.375. Overall, OAs outperformed YAs, regardless of whether feedback was provided. Correct responses to DR items exceeded those to DK items – even in the absence of feedback. As evident in the Age by Response interaction, OAs appeared to be better than YAs at correctly marking an item initially as DR (an accessibility-related retrieval failure) versus DK (an availability-related retrieval failure). A notable point is that OAs appear to be better than YAs at making use of the feedback provided, as demonstrated by their higher final test accuracy. Although interpreting a null effect is done with caution, the lack of a 3-way interaction does suggest that, with or without feedback, OAs are succeeding in the task. As noted in the introduction, the greater success observed in OAs over YAs could have reflected a metacognitive error; however, items classified as DK were, in fact, not recovered, as evidenced by the very low rates of correct responses on the final test.

One of our core questions Experiment 3 allowed us to address was whether *not remembered* items would result in higher rates of recovery (i.e., correct responses) than items *not known* specifically in the absence of feedback. The results of Experiment 1, in which no feedback was provided, are consistent with this; however, the final multiple-choice test differs from the present short answer test in the amount of retrieval effort. Because on a multiple-choice test the correct answer is presented among foils, there is less demand on an active search through memory and greater contributions of familiarity-based responding. Indeed, the foils may act as useful cues toward the correct answer. Therefore, it is not clear to what extent performance on a multiple-choice test truly reflects spontaneous recovery of previously inaccessible information.

To explore this directly, we examined the rates of spontaneous recovery that are able to be observed in the No Feedback condition. That is, we excluded initially correct and incorrect items to focus on retrieval failures (i.e., items deemed to be unavailable or inaccessible in the No Feedback group). If both OAs and YAs perform better on DR than DK items, this would suggest similar rates of spontaneous recovery. However, if OAs spontaneously recover more marginal knowledge than YAs, as suggested by prior work (Umanath, 2016) they would be expected to correctly answer more DR questions than YAs. Importantly, if the same pattern (better performance for OAs than YAs) occurs for DK responses, then OAs’ performance on these items in earlier studies was potentially due, at least in part, to metacognitive errors or to more effective encoding and retrieval of correct answer feedback rather than to recovery and therefore, fluctuating access to the knowledge base. Therefore, we submitted correct responses on the final test from the No Feedback condition to a 2 (Initial response: DR vs. DK) × 2 (Age) mixed ANOVA. Data from 40 OAs and 39 YAs were included. OAs (*M* = 0.06, SE = 0.01) outperformed YAs (*M* = 0.02, SE = 0.01), *F*(1, 77) = 8.60, *p* = 0.004, *η*_p_^2^ = 0.10. Consistent with Experiments 1 and 2, DR items (*M* = 0.06, SE = 0.01) were retrieved more accurately than DK items (*M* = 0.02, SE = 0.005), *F*(1, 77) = 21.11, *p* < 0.001, *η*_p_^2^ = 0.22. Critically, the interaction was significant, *F*(1, 77) = 10.31, *p* = 0.002, *η*_p_^2^ = 0.12. YAs’ accuracy did not differ as a function of initial response, *F*(1, 77) = 0.95, *p* = 0.334, *η*_p_^2^ = 0.01, suggesting little to no recovery of knowledge under conditions of no feedback, regardless of whether YAs deemed these items inaccessible or unavailable. In contrast, OAs *did* successfully recover more items given a DR response than those given a DK response, *F*(1, 77) = 30.85, *p* < 0.001, *η*_p_^2^ = 0.29. In sum, even in the absence of correct answer feedback for questions that were quite difficult, OAs do recover some—significantly more than for the initial DK items and more so than YAs. Recovery of DK items was essentially at floor for both age groups. That this difference is significant is meaningful, though it is based on a small number of items.

Thus, OAs did recover answers to items they indicated were temporarily inaccessible more than YAs. Moreover, OAs’ recovery of DK items was significantly lower and indeed, essentially at floor. Combined, these results suggest that OAs do have preserved metacognitive awareness, and the repeated advantage observed for DK items on the final test is unlikely to be due primarily to a metacognitive error of underestimating their knowledge. Instead, it is more likely to be due, in part, to more effective encoding and memory of the correct answer feedback as well as fluctuations in access to their knowledge base. We also note, briefly, that the overall distribution of responses and means for online participants were remarkably similar to those obtained in the laboratory.

## General discussion

### Maintenance of metacognitive distinction between DR as inaccessible and DK as unavailable

First, both OAs and YAs demonstrated the ability to distinguish between retrieval failures due to a lack of accessibility versus availability when retrieval failures were quite frequent due to the difficulty of the materials. Thus, the present work replicates and, importantly, extends the findings of Coane and Umanath (2019). Interestingly, changing the baseline accessibility of the items resulted in a similar assessment process but with more extreme differences than observed previously. Phenomenological judgments of inaccessibility and unavailability do seem to involve a relative/comparative process, where participants anchor their responses around some judgment of what “feels” inaccessible vs. unavailable. Critically, the DR/DK distinction remained valid for both age groups and was still successfully applied intuitively by capitalizing on natural language use. Ultimately, the findings support the idea that OAs’ metacognitive awareness of their knowledge bases is maintained across different distributions of knowledge accessibility.

### Effective use of feedback

The results from Experiments 2 and 3 demonstrate that there are circumstances under which OAs can use correct answer feedback more effectively than YAs. When provided with correct answer feedback after retrieval failures on the initial short-answer test, OAs, both in the lab and online, answered more questions correctly on the final test compared to YAs. This is consistent with prior work showing that not only do OAs benefit from corrective feedback; in some cases, they benefit *more* than YAs, at least in the context of general knowledge. Specifically, OAs often show a smaller hypercorrection effect (greater correct of high confidence errors compared to low confidence ones) than YAs, which might reflect poorer encoding of feedback (Cyr and Anderson, 2013; Eich et al., 2013). However, as demonstrated by Metcalfe et al. (2015), the reduced hypercorrection effect is actually due to the fact that OAs correct more low confidence errors than YAs—in other words, they corrected more errors overall. Furthermore, in the same study, OAs appeared to learn more than YAs from the feedback for items that they indicated were unfamiliar. OAs can also successfully retrieve their original error and correct it and, interestingly, OAs corrected more errors than YAs when they could not recall their original errors and maintained the corrected information longer than YAs (Sitzman et al., 2020). Similarly, in the present work, for initial-Incorrect items, OAs were better able to correct their mistakes than YAs.

For initial-DK items, which are supposed to represent information that participants believe is unavailable or was never stored in their memory, OAs made better use of the correct answer feedback than did YAs. Given that OAs tend to struggle to learn new information compared to YAs (Balota et al., 2000), prior research would suggest that feedback on the initial test would not have been as useful to them. However, perhaps due to their extensive knowledge bases, OAs may have had some accessible related content that would elicit motivation or value in learning (Castel, 2007) or simply facilitate integration of new associated information (e.g., Schustack and Anderson, 1979; Kole and Healy, 2007), making effective use of the presented feedback for correcting errors and/or learning seemingly new information. Regarding the hyper-correction effect, high-confidence errors could also be indicators of marginal knowledge where participants are willing to hazard a guess. Indeed, both age groups are more likely to indicate that they actually “knew it all along” following higher confidence errors (Metcalfe and Finn, 2011; Sitzman et al., 2015). This would suggest that effective use of feedback may not always be new learning and include a strong episodic component; it may just be strengthening access to marginal knowledge. Future work involving confidence will be useful to further understanding OAs’ effective use of feedback.

### Recovery of marginal knowledge

OAs recovered access to more marginal knowledge than did YAs. This can be observed both when OAs did and did not receive correct answer feedback. For initial-DR responses, which represent marginal knowledge or knowledge that is thought to be temporarily inaccessible, OAs answered more questions correctly on the subsequent final test, regardless of the form of that final test. OAs’ performance is typically facilitated by greater environmental support (Craik, 1983, 1986), which multiple-choice tests provide relative to short-answer tests. Yet here, a single retrieval attempt of general knowledge with feedback (Experiments 2 & 3) allowed OAs to produce the information in a (more difficult) recall task just as well as they recognized it on a recognition test (Exp. 1). Fundamentally, the correct answer feedback in Experiments 2 and 3 served as a more effective reminder for OAs than for YAs. This finding extends the effectiveness of feedback for stabilization of marginal knowledge previously found for YAs (Berger et al., 1999) to OAs, albeit likely based on different underlying mechanisms.

Why might OAs recover more marginal knowledge than YAs? Their perseverant recovery of knowledge could simply be due to the (larger) size of their knowledge bases (Gollan and Brown, 2006), making it more difficult to quickly access any particular content, akin to the fan effect (Anderson, 1974). A number of recent studies have focused on the role of curiosity in the processing of feedback (Bloom et al., 2018; Metcalfe et al., 2022). Specifically, when an error is made, individuals tend to be more curious about the correct answer, and this effect is magnified when participants are in a TOT state. In the present context, DR responses presumably reflected both TOT states as well as less imminent retrieval states or less accessible knowledge; this would elicit greater curiosity to know the answer and lead to processing of the feedback. DK items, because they are not associated with a sense of imminent or even possible retrieval, might elicit less curiosity and therefore, the feedback would be processed less deeply. Because of their more extensive knowledge bases, OAs not only responded correctly more often than YAs, they also used DR more and provided more incorrect responses. Therefore, they likely experienced greater curiosity, which enabled them to successfully integrate the feedback.

A more common explanation would be that OAs were mistaken in marking items as DK when they were actually marginal knowledge and in the same way as for the initial-DR items, the feedback served as a reminder. That is, OAs made a metacognitive error about what was unavailable versus inaccessible in memory. Yet, the evidence does not support this explanation. Notably, in the absence of feedback on the initial short-answer test in Experiment 3, OAs still recovered access to *more* marginal knowledge than YAs. In fact, younger adults failed to recover marginal knowledge when no feedback was provided, showing no difference in correct answers on the final test for initial-DR versus -DK responses. Critically, in contrast, OAs answered more initial-DR questions correctly than initial-DK questions, showing spontaneous recovery of access consistent with prior work (Cohen and Faulkner, 1986; Burke et al., 1991; Umanath, 2016). Thus, OAs were unlikely to have been making metacognitive errors in the previous studies and incorrectly labeling marginal knowledge as DK or unavailable. In conjunction with the evidence from the Feedback condition, these results indicate that not only do OAs accurately assess whether information is unavailable or inaccessible; they are also very likely learning effectively from the feedback. We acknowledge that it remains possible that even still, some initial-DK responses could refer to marginal knowledge that is especially difficult to access. However, we would hazard to say that perhaps such information that is so hard to access essentially behaves like new or unavailable information.

### Conclusions and future directions

Although we demonstrated compellingly that both YA and OAs can and do differentiate between the states of *not remembering* and *not knowing*, the present work is not without limitations. First and foremost, the materials are squarely within the realm of semantic/crystallized knowledge, so the extent to which these findings generalize to materials more dependent on episodic memory systems is unclear (but see Lukasik et al., 2020; Umanath et al., 2023). Second, we did not collect confidence ratings, which limits our ability to examine to what extent errors are corrected as a function of confidence. Third, the final tests occurred only after a short delay. Though there is evidence that OAs maintain memory of corrective feedback (Sitzman et al., 2020), exploring the durability of this new learning would be beneficial and shed light on integration of information into their knowledge bases. Finally, we acknowledge that we did not explicitly manipulate difficulty in the present work; therefore, although we can confidently state that appropriate use of DR and DK is preserved with difficult materials, we cannot draw firm conclusions about the direct impact of difficulty.

Additionally, there are several avenues for future work in order to further bolster the findings here that OAs’ high rates of access recovery after initially providing DK responses are not reflective of a metacognitive mis-calibration. One way to do this might be to couple a phenomenological approach with ratings of confidence as is often done in the metacognition literature (e.g., Dunlosky et al., 2005; Hertzog et al., 2013). This would also allow for examination of hypercorrection effects in the context of *not remembering* versus *not knowing*. Another approach could be to further characterize phenomenological experiences of retrieval failures from TOTs to varying levels of inaccessibility to unavailability. Though DR and DK do distinguish the two states broadly, each may still encompass a range of cognitive states of different material in the knowledge base with differential consequences for recovery and new learning.

Together, the findings indicate that, although both younger and older adults’ metacognitive awareness regarding underlying causes of retrieval failures is maintained across different distributions of knowledge accessibility, OAs use correct answer feedback more effectively than YAs. Importantly, in the absence of feedback, OAs can spontaneously recover marginal knowledge, whereas YAs do not. That OAs have larger knowledge bases than YAs is robustly well established, but these findings are remarkable in the broader context of the memory and aging literature and add to a burgeoning field of work showing positive aspects of cognition in aging. It is worth noting that remembering the correct answer feedback after *not knowing* is likely reflective of some new episodic learning – something OAs have consistently been reported to have difficulties with. Here, with general knowledge questions, we show remarkable preservation of this ability among others– ones that enable OAs to outperform their younger counterparts.

## Data availability statement

All stimuli and data are available on JHC’s website: https://web.colby.edu/memoryandlanguagelab/publications/stimuli-and-data-sets/.

## Ethics statement

The studies involving human participants were reviewed and approved by Institutional Review Board at Colby College and Claremont McKenna College. The patients/participants provided their informed consent to participate in this study.

## Author contributions

SU: conceptualization, methodology, writing, reviewing, and editing. JC: conceptualization, data curation, analyses, writing, editing, reviewing, and funding acquisition. TB, SK, and CW: stimulus development, programming, data collection, and writing. All authors contributed to the article and approved the submitted version.

## Funding

This work was supported by funding from a James McDonnell Foundation Understanding Human Cognition Grant awarded to JHC (#220020426). The funding agency had no input on the study design, data analysis, or writing.

## Conflict of interest

The authors declare that the research was conducted in the absence of any commercial or financial relationships that could be construed as a potential conflict of interest.

## Publisher’s note

All claims expressed in this article are solely those of the authors and do not necessarily represent those of their affiliated organizations, or those of the publisher, the editors and the reviewers. Any product that may be evaluated in this article, or claim that may be made by its manufacturer, is not guaranteed or endorsed by the publisher.

## Footnotes

^1^ https://www.prolific.co/

^2^ www.gorilla.sc

^3^ https://www.prolific.co/
